# A supporting ecosystem to mature extracellular vesicles into clinical application

**DOI:** 10.15252/embj.2018101412

**Published:** 2019-04-12

**Authors:** Olivier De Wever, An Hendrix

**Affiliations:** ^1^ Laboratory of Experimental Cancer Research Department of Human Structure and Repair Ghent University Ghent Belgium; ^2^ Cancer Research Institute Ghent Ghent Belgium

**Keywords:** Membrane & Intracellular Transport

## Abstract

Research into extracellular vesicles (EV) has yielded important biological insights and raised the prospect of developing novel diagnostics and therapeutics for a wide range of pathologies. As with other emerging and transformative fields in research, it will require a broad, supportive base for EV research to mature and to develop clinical application. Here, we identify several focus areas to further improve reproducibility and reliability specifically for EV research and make recommendations for minimal experimental guidelines, transparency tools, reference materials, validation, identification of contaminants, data sharing, coaching through education, and funding opportunities.

## Extracellular vesicle research and applications

Extracellular vesicles (EV) are membrane‐enclosed nanoparticles that contain proteins, nucleotides, lipids and metabolites. Eukaryotic cells secrete EV through orchestrated plasma membrane budding or fusion of multi‐vesicular endosomes with the plasma membrane. A third mechanism of EV genesis takes place during controlled cell death when cells fragment into apoptotic bodies (van Niel *et al*, 2018). EV are not unique to eukaryotic cells: both Gram^+^ and Gram^−^ bacteria can release EV by outward budding of the prokaryotic membrane (Toyofuku *et al*, 2019). EV have been attributed biological functions in physiology and diseases such as cancer and cardiovascular, neurological, and immune‐related disorders. Although these vesicles were discovered more than 40 years ago, it was only during the past decade that scientists began to understand their function in cell‐to‐cell communication on the molecular level and their role in physiology and pathology.

Extracellular vesicles have been identified in any human biofluid, including blood, and therefore offer a possibility for easy and efficient diagnosis and monitoring of disease progression—potential applications in cancer diagnostics are one example of imminent use. Bulk EV isolated from blood followed by glypican‐1 protein single‐marker analysis can identify early‐stage pancreatic cancer; EV subtypes isolated using antibody cocktails that target EV surface proteins showed higher specificity for pancreatic cancer compared to single‐marker isolation. Similarly, EV hold great potential for developing new therapies or delivery systems for existing drugs. EV from selected eukaryotic and prokaryotic cell types have already been used as therapeutic agents in oncology, regenerative medicine, and for vaccination. As a vehicle for drug delivery, siRNA‐loaded EV show remarkable efficacy in treating multiple animal models of cancer.

Given their potential for diagnosis and therapy, EV‐related research and applications have attracted considerable commercial interest and investment. The number of EV‐related patents has been increasing steadily during the past decade, and the production of EV at therapeutically relevant quantities and with good manufacturing practices (GMP) is underway. The global market for EV‐based diagnostics and therapeutics is projected to grow from US$25 million in 2018 to US$180 million in 2023—a 5‐year compound annual growth rate (CAGR) of 48.4% (https://www.bccresearch.com/market-research/biotechnology/exosome-diagnostics-and-therapeutics-global-markets-report-bio149b.html).

Although these vesicles were discovered more than 40 years ago, it was only during the past decade that scientists began to understand their function in cell‐to‐cell communication on the molecular level and their role in physiology and pathology.

However, the plethora of methods to separate and characterize EV, the intrinsic heterogeneity of EV subtypes with varying size (from 40 to > 500 nm), molecular patterns (Tkach *et al*, 2017), their origin (Tulkens *et al*, 2018), and the complexity of biofluids present considerable challenges for rigorous and reproducible research as a basis for clinical applications (Van Deun *et al*, 2017). We therefore discuss potential areas of concern and propose recommendations to cope with these problems and challenges. To be useful, recommendations must be widely embraced by the community, which requires further discussion and advice. This commentary will hopefully encourage said discussion and provide useful steps for transforming fundamental EV research into clinical applications.

## Situating the areas of concern

There is no one‐size‐fits‐all method for analyzing EV. Biofluids are complex mixtures that contain many components some of which share biochemical and physical characteristics with EV (Simonsen, 2017). Different methods that separate EV with variable purity are used and identify method‐dependent functions or biomarkers. Some separation methods have low selectivity and yield multiple EV subtypes, while others are more selective to include or exclude specific EV subtypes. Some biophysical characterization methods are not able to measure smaller sized EV subtypes and thereby underestimate EV numbers, whereas others fail to discriminate between EV and contaminants—ribo‐ or lipoproteins and other aggregates—and potentially overestimate the quantity of EV. Additionally, unlike inorganic metal nanoparticles that generally have the same size in the “wet” and “dry” states, EV can undergo substantial size changes, depending on the analysis method used—for instance, electron microscopy (dry state) versus hydrodynamic radius calculation from dynamic light scattering analysis (wet state). As such, interpretation of EV data remains far from easy and straightforward and requires proper training and experience of researchers but also reviewers and editors.

The complex composition of biofluids requires branched development of complementary separation and characterization methods to unlock the secrets of EV. Comparing methods and transparency in reporting experimental parameters will be indispensable to direct and understand this development. Methodological comparison and cross‐laboratory studies also require reference materials to deal with technical variability and reveal biological differences. Transparency is necessary to improve the exchange of (meta)data and allow systematic comparison between approaches.

## A supporting ecosystem based on eight pillars

**Figure 1:**
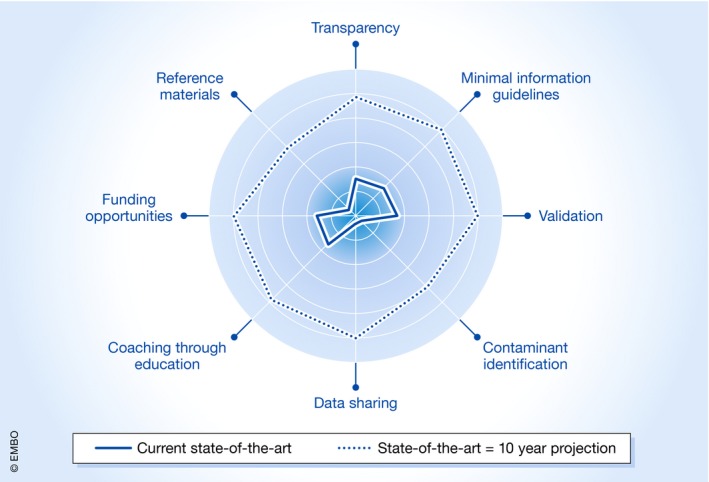
**Radar plot depicting eight pillars of a supporting ecosystem to mature EV into clinical application**.

The EV research community has already started initiatives to cope with some of these challenges (Van Deun *et al*, 2017). However, the most important missing element is that all areas of concern are connected and interdependent. Dealing with one or two of these individually will not be sufficient to move the field forward. It is their complementary and combined aspect that will lead to synergy and changes. We therefore propose a supporting ecosystem based on eight pillars to make the field aware of critical issues and present solutions (Fig 1).

### Minimal information guidelines

There are established guidelines for selecting and interpreting separation and characterization methods for EV; these are useful for researchers as well as reviewers who need to provide realistic and reasonable critique of papers focused on EV. These guidelines were first published in 2014 and updated in 2018. Minimal information guidelines are intertwined with experience‐based advice and position papers with a focus on biofluid collection, storage, and use for EV analysis, clinical grade EV preparation, and EV‐associated RNA analysis. Importantly, guidelines should be focused on parameters and technologies that are accessible to most research teams and receive broad consensus, which is a necessity for broad implementation.

### Transparency and tools to promote transparency

The plethora of methods and protocols can be confusing, but it is probably unavoidable, given the diverse nature of EV and innovative character of research. In many cases, scientists find that their exact requirements are not met by current tools and often have to create custom solutions. Protocols should therefore be open and transparent, so that all scientists can understand them, recognize their value, and can adhere to them, even if the technology advances—as will be the case.

To cope with this variety of methods, the community needs to be aware of essential experimental parameters and to report them so anyone can understand each experiment and reproduce the results. Specifically, the EV‐TRACK knowledgebase (Van Deun *et al*, 2017), an online open‐access resource to track and organize data on EV separation and characterization, is therefore suitable to monitor progress in the field in a standardized format. The current set‐up includes a checklist of nine essential experimental parameters that are bundled into an EV‐METRIC (http://evtrack.org/) to improve transparency. Interpretation of reported data would be further improved by using Research Resource Identifiers (RRID) to identify key resources, that is, reagents, tools, and materials (https://scicrunch.org/resources). RRID meet three key criteria: they are machine‐readable, free to generate and access, and are consistent across publishers and journals.

The complex composition of biofluids requires branched development of complementary separation and characterization methods to unlock the secrets of EV.

Admittedly, both the implementation of checklists (such as EV‐METRIC) and RRID may be time‐consuming for authors, but it will improve reporting of essential experimental parameters and unambiguous resource identification, two crucial aspects for increasing reproducibility. In addition, the EV‐TRACK knowledgebase, as it is available to the whole community, could fundamentally shift our interaction with the literature and may facilitate protocol design and reassessment and reuse of published data. No doubt, there will be challenges to incorporate EV‐TRACK into large‐scale practice. A main aspect is the available resources to upload the massive EV‐related data (currently more than 1,000 research papers/year). Uploading could be done by one (or more) of many parties: authors, journal staff, third‐party scientists, or even algorithms. Prospective manual upload is feasible when authors submit a paper for peer review. However, manual upload is difficult retrospectively given the massive number of EV‐related papers already published. Automated, artificial intelligence‐driven approaches may come to the rescue. Finally, the performance of the EV‐TRACK pipeline itself would need to be evaluated.

### Reference materials for normalization or calibration

Biological reference materials are available but are currently not or poorly trackable and are indistinguishable from sample EV. Synthetic reference materials, such as silica beads, are particularly useful for the calibration of optical EV detection methods, but have poor application for normalization because they do not have a wide size distribution, surface and luminal biomolecules, and different density characteristics. Ideally, EV biomimetics, with similar size, density, and biochemical composition but easily distinguishable from sample EV (e.g., through a fluorescent probe), should be available for research. Major challenges for biological reference EV are their large complicated macromolecular structure; their heterogeneity; and their complicated production and purification. EV reference materials must also come in narrow size ranges to calibrate characterization methods and to evaluate size specificity of separation methods. Efforts should be stimulated to design robust control reference materials by academics, and commercial and quality assurance program providers. Their subsequent promotion is crucial to obtain maximum understanding and impact, and to facilitate consistent application.

### Validation: comparison of separation and characterization methods, interlaboratory studies, and benchmarking of novel methods

Several technologies have been developed to separate or measure EV, but there are no appropriate and quantifiable performance metrics for these technologies, which hampers informed selection of the most appropriate method for the particular study objectives. Studies are needed to objectively assess technology performance in terms of repeatability, sensitivity, accuracy, specificity, and efficiency. Interlaboratory evaluation to assess reproducibility and benchmarking of novel methods is of great importance before widespread use or clinical implementation. The International Society for Extracellular Vesicles (ISEV) and a number of national EV societies may stimulate researchers to perform validation studies. We consider the establishment of EV core facilities (https://www.helsinki.fi/en/researchgroups/extracellular-vesicles/ev-core) and the use of automated liquid handlers as important contributors to facilitate this interlaboratory validation and reproducibility.

### Identification of true contaminants versus true content

Generally, a short list of EV contaminant proteins, lipids, or RNA species is not available to the community. Although this knowledge is essential for a sensitive and selective use of EV in clinics and although efforts are underway to compile such a list they are not yet widely implemented as evidenced from EV‐TRACK data. Owing to the vesicular nature of EV, true content is more easily identified as transmembrane or GPI‐anchored proteins or cytosolic proteins with lipid or membrane protein‐binding ability. No such data are available for other classes of proteins, lipids, or nucleotides.

Another and poorly described source of complexity is the likely presence of a corona of various molecules on the EV surface. In particular, plasma proteins, such as complement factors, immunoglobulins, or lipoproteins, are frequently reported to be part of a corona that may also involve nucleotides. Knowing that some methods may co‐isolate true contaminants while other methods cause the loss of true content in the form of corona proteins/nucleotides, progress in this area may be the most challenging for the years ahead.

### (meta)data sharing and annotation

Vesiclepedia (http://www.microvesicles.org) is a web‐based compendium of proteins, RNA, lipids, and metabolites that are identified in EV (Kalra *et al*, 2012). Studies in Vesiclepedia are currently annotated with an EV‐METRIC for transparent reporting on EV isolation and characterization methods (Pathan *et al*, 2018). This is a major improvement, because it informs the end user about certain standards in EV separation characterization methods and allows stringent searches can be performed. Yet, the data sets (protein–RNA–lipid–sugars–metabolites) generated in EV research currently remain isolated owing to the lack of a data‐sharing system. In addition, raw images and videos—for example, electron microscopy and nanoparticle tracking analysis (NTA)—are generally not reported in publications and remain an untapped potential for data reanalysis. Thus, a centralized data repository system combined with knowledge of experimental parameters (EV‐TRACK annotation) will allow novel approaches to maximize the utility of the collected data.

### Coaching through training and education

Hands‐on courses and didactic programs for early‐stage and experienced researchers by educational days or workshops is a recent focus to advance education and experience (https://www.embl.de/training/events/2019/EXO19-01/; https://www.isev.org/page/ISEV2018Education; https://www.isev.org/page/ChinaWorkshop).

These courses, combined with transparency tools, will help to optimize methodological rigor and reproducibility in EV research. Although we realize that instructor‐led training would be limited (hands‐on EMBL courses reach 25 persons/year; ISEV educational days reach ±2,000 persons/year), online videos (YouTube, JoVE) or massive open online courses (https://www.coursera.org/learn/extracellular-vesicles) may greatly enhance the target audience. As transparency is an issue in all scientific fields, the NIH provides free online educational material as part of a training initiative (https://www.nih.gov/research-training/rigor-reproducibility/training#Modules). Such efforts should be incorporated into basic scientific education and resources such as the European Open Science Cloud.

Although governments and national and international EV societies play an essential role in didactic programs and training, each principal investigator should take responsibility as well. For example, laboratory‐based journal clubs are invaluable training formats that allow discussion of science, methodology, and transparency in the context of a specific publication and strongly influence the perception of a publication by young and experienced researchers.

### Funding opportunities to stimulate benchmarking and interlaboratory studies

Scientific rigor is the strict application of the scientific method to ensure unbiased and well‐controlled experimental design, methodology, analysis, interpretation, and reporting of results. Both the United States and Europe started funding opportunities to promote rigor, reproducibility, and metrology in EV isolation, characterization, and computational analysis (https://grants.nih.gov/grants/guide/pa-files/PAR-16-277.html; https://msu.euramet.org/calls.html). Although any efforts to improve reproducibility will require a measured investment in capital and time, the long‐term benefits to society derived from increased scientific fidelity will greatly exceed the upfront costs (Freedman *et al*, 2015).

## Conclusion

The eight actionable suggestions outlined above provide a supporting ecosystem to increase rigor and reproducibility in EV research. As in each ecosystem, these pillars are interdependent. Reference materials and validation experiments will support identification of contaminants. This knowledge will be implemented in guidelines that mandate transparent reporting for data sharing. The community needs to be coached and educated about the areas of concern and respective solutions. Financial support is a prerequisite to move the field forward.

This ecosystem will ensure a steady supply of innovative, reliable, and reproducible discoveries to translate EV research into societal benefits. Early clinical successes with bacterial‐derived EV as a vaccination strategy raise tremendous hopes that EV may indeed reach wide clinical application. The road toward success will come more rapidly with the full engagement of the entire EV research community.

## Conflict of interest

The authors declare that they have no conflict of interest.

**Further reading**

**Diagnostic use of EV**

Melo SA, Luecke LB, Kahlert C, Fernandez AF, Gammon ST, Kaye J, LeBleu VS, Mittendorf EA, Weitz J, Rahbari N, Reissfelder C, Pilarsky C, Fraga MF, Piwnica‐Worms D, Kalluri R (2015) Glypican‐1 identifies cancer exosomes and detects early pancreatic cancer. *Nature* 523: 177–182

Yang KS, Im H, Hong S, Pergolini I, Del Castillo AF, Wang R, Clardy S, Huang C‐H, Pille C, Ferrone S, Yang R, Castro CM, Lee H, Del Castillo CF, Weissleder R (2017) Multiparametric plasma EV profiling facilitates diagnosis of pancreatic malignancy. *Sci Transl Med* 9: eaal3226

**Therapeutic use of EV**

Besse B, Charrier M, Lapierre V, Dansin E, Lantz O, Planchard D, Le Chevalier T, Livartoski A, Barlesi F, Laplanche A, Ploix S, Vimond N, Peguillet I, Théry C, Lacroix L, Zoernig I, Dhodapkar K, Dhodapkar M, Viaud S, Soria J‐C, *et al* (2016) Dendritic cell‐derived exosomes as maintenance immunotherapy after first line chemotherapy in NSCLC. *Oncoimmunology* 5: e1071008

Dai S, Wei D, Wu Z, Zhou X, Wei X, Huang H, Li G (2008) Phase I clinical trial of autologous ascites‐derived exosomes combined with GM‐CSF for colorectal cancer. *Mol Ther* 16: 782–790

Kamerkar S, LeBleu VS, Sugimoto H, Yang S, Ruivo CF, Melo SA, Lee JJ, Kalluri R (2017) Exosomes facilitate therapeutic targeting of oncogenic KRAS in pancreatic cancer. *Nature* 546: 498–503

Kordelas L, Rebmann V, Ludwig A‐K, Radtke S, Ruesing J, Doeppner TR, Epple M, Horn PA, Beelen DW, Giebel B (2014) MSC‐derived exosomes: a novel tool to treat therapy‐refractory graft‐versus‐host disease. *Leukemia* 28: 970–973

Petousis‐Harris H, Paynter J, Morgan J, Saxton P, McArdle B, Goodyear‐Smith F, Black S (2017) Effectiveness of a group B outer membrane vesicle meningococcal vaccine against gonorrhoea in New Zealand: a retrospective case‐control study. *Lancet* 390: 1603–1610

Pi F, Binzel DW, Lee TJ, Li Z, Sun M, Rychahou P, Li H, Haque F, Wang S, Croce CM, Guo B, Evers BM, Guo P (2018) Nanoparticle orientation to control RNA loading and ligand display on extracellular vesicles for cancer regression. *Nat Nanotechnol* 13: 82–89

**Minimal information guidelines and position papers concerning EV**

Coumans FAW, Brisson AR, Buzas EI, Dignat‐George F, Drees EEE, El‐Andaloussi S, Emanueli C, Gasecka A, Hendrix A, Hill AF, Lacroix R, Lee Y, van Leeuwen TG, Mackman N, Mäger I, Nolan JP, van der Pol E, Pegtel DM, Sahoo S, Siljander PRM, *et al* (2017) Methodological guidelines to study extracellular vesicles. *Circ Res* 120: 1632–1648

Gimona M, Pachler K, Laner‐Plamberger S, Schallmoser K, Rohde E (2017) Manufacturing of human extracellular vesicle‐based therapeutics for clinical use. *Int J Mol Sci* 18: 1190

Lener T, Gimona M, Aigner L, Börger V, Buzas E, Camussi G, Chaput N, Chatterjee D, Court FA, Del Portillo HA, O'Driscoll L, Fais S, Falcon‐Perez JM, Felderhoff‐Mueser U, Fraile L, Gho YS, Görgens A, Gupta RC, Hendrix A, Hermann DM, *et al* (2015) Applying extracellular vesicles based therapeutics in clinical trials—an ISEV position paper. *J Extracell Vesicles* 4: 30087

Théry C, Witwer KW, Aikawa E, Alcaraz MJ, Anderson JD, Andriantsitohaina R, Antoniou A, Arab T, Archer F, Atkin‐Smith GK, Ayre DC, Bach J‐M, Bachurski D, Baharvand H, Balaj L, Baldacchino S, Bauer NN, Baxter AA, Bebawy M, Beckham C, *et al* (2018) Minimal information for studies of extracellular vesicles 2018 (MISEV2018): a position statement of the International Society for Extracellular Vesicles and update of the MISEV2014 guidelines. *J Extracell Vesicles* 7: 1535750

Witwer KW, Buzás EI, Bemis LT, Bora A, Lässer C, Lötvall J, Nolte‐’t Hoen EN, Piper MG, Sivaraman S, Skog J, Théry C, Wauben MH, Hochberg F (2013) Standardization of sample collection, isolation and analysis methods in extracellular vesicle research. *J Extracell Vesicles* 2: 20360

Reiner AT, Witwer KW, van Balkom BWM, de Beer J, Brodie C, Corteling RL, Gabrielsson S, Gimona M, Ibrahim AG, de Kleijn D, Lai CP, Lötvall J, Del Portillo HA, Reischl IG, Riazifar M, Salomon C, Tahara H, Toh WS, Wauben MHM, Yang VK, *et al* (2017) Concise review: developing best‐practice models for the therapeutic use of extracellular vesicles. *Stem Cells Transl Med* 6: 1730–1739

Mateescu B, Kowal EJK, van Balkom BWM, Bartel S, Bhattacharyya SN, Buzás EI, Buck AH, de Candia P, Chow FWN, Das S, Driedonks TAP, Fernández‐Messina L, Haderk F, Hill AF, Jones JC, Van Keuren‐Jensen KR, Lai CP, Lässer C, di Liegro I, Lunavat TR, *et al* (2017) Obstacles and opportunities in the functional analysis of extracellular vesicle RNA—an ISEV position paper. *J Extracell Vesicles* 6: 1286095

**Technical complexity of EV research**

Buzás EI, Tóth EÁ, Sódar BW, Szabó‐Taylor KÉ (2018) Molecular interactions at the surface of extracellular vesicles. *Semin Immunopathol* 40: 453–464

Faria M, Björnmalm M, Thurecht KJ, Kent SJ, Parton RG, Kavallaris M, Johnston APR, Gooding JJ, Corrie SR, Boyd BJ, Thordarson P, Whittaker AK, Stevens MM, Prestidge CA, Porter CJH, Parak WJ, Davis TP, Crampin EJ, Caruso F (2018) Minimum information reporting in bio‐nano experimental literature. *Nat Nanotechnol* 13: 777–785

Gardiner C, Shaw M, Hole P, Smith J, Tannetta D, Redman CW, Sargent IL (2014) Measurement of refractive index by nanoparticle tracking analysis reveals heterogeneity in extracellular vesicles. *J. Extracell Vesicles* 3: 25361

Maas SLN, de Vrij J, van der Vlist EJ, Geragousian B, van Bloois L, Mastrobattista E, Schiffelers RM, Wauben MHM, Broekman MLD, Nolte‐’t Hoen ENM (2015) Possibilities and limitations of current technologies for quantification of biological extracellular vesicles and synthetic mimics. *J Control Release* 200: 87–96

Monopoli MP, Aberg C, Salvati A, Dawson KA (2012) Biomolecular coronas provide the biological identity of nanosized materials. *Nat Nanotechnol* 7: 779–786

Schöttler S, Becker G, Winzen S, Steinbach T, Mohr K, Landfester K, Mailänder V, Wurm FR (2016) Protein adsorption is required for stealth effect of poly(ethylene glycol)‐ and poly(phosphoester)‐coated nanocarriers. *Nat Nanotechnol* 11: 372–7

Valkonen S, van der Pol E, Böing A, Yuana Y, Yliperttula M, Nieuwland R, Laitinen S, Siljander PRM (2017) Biological reference materials for extracellular vesicle studies. *Eur J Pharm Sci* 98: 4–16

Van Deun J, Mestdagh P, Sormunen R, Cocquyt V, Vermaelen K, Vandesompele J, Bracke M, De Wever O, Hendrix A (2014) The impact of disparate isolation methods for extracellular vesicles on downstream RNA profiling. *J Extracell Vesicles* 3: 24858

Varga Z, van der Pol E, Pálmai M, Garcia‐Diez R, Gollwitzer C, Krumrey M, Fraikin J‐L, Gasecka A, Hajji N, van Leeuwen TG, Nieuwland R (2018) Hollow organosilica beads as reference particles for optical detection of extracellular vesicles. *J Thromb Haemost* 16: 1646–1655
